# TIES 2.0: A Dual-Topology Open Source Relative Binding Free Energy Builder with Web Portal

**DOI:** 10.1021/acs.jcim.2c01596

**Published:** 2023-01-31

**Authors:** Mateusz K. Bieniek, Alexander D. Wade, Agastya P. Bhati, Shunzhou Wan, Peter V. Coveney

**Affiliations:** † Centre for Computational Science, Department of Chemistry, University College London, London WC1H 0AJ, United Kingdom; ‡ School of Natural and Environmental Sciences, Newcastle University, Newcastle upon Tyne NE1 7RU, United Kingdom; ¶ Advanced Research Computing Centre, University College London, London WC1H 0AJ, United Kingdom; § Institute for Informatics, Faculty of Science, University of Amsterdam, 1098XH Amsterdam, The Netherlands

## Abstract

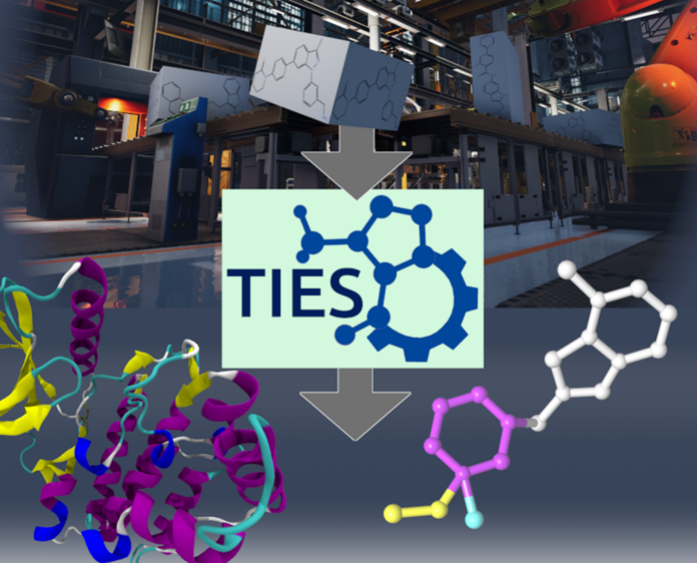

Relative binding free energy (RBFE) calculations are widely used to aid the process of drug discovery. TIES, Thermodynamic Integration with Enhanced Sampling, is a dual-topology approach to RBFE calculations with support for NAMD and OpenMM molecular dynamics engines. The software has been thoroughly validated on publicly available datasets. Here we describe the open source software along with a web portal (https://ccs-ties.org) that enables users to perform such calculations correctly and rapidly.

## Introduction

Free energy (FE) calculations comprise a family of physics-based approaches used to compute the difference in energies between different thermodynamic states. The most common approaches to FE calculations can be largely divided into two groups: “end-point” and alchemical methods. The “end-point” methods such as MM(GB/PB)SA^1−3^ estimate the binding energy by comparing the energy of the complex to the energy of the constituent elements. Alchemical methods track the changes in energy along a fictitious pathway between two slightly different molecules. The transformation between the two molecules is referred to as “alchemical”. The simplest example of such a transformation (or “transmutation”) is an atom mutation. In the context of ligand–protein binding, FE calculations are used to quantify the strength of the binding to determine, for example, if a ligand has a higher potency for the desired protein target, or if it has fewer off-target interactions. The relative binding free energy (RBFE) method is one of the most successful approaches to FE calculations. In order for the method to be valid the pair of ligands used must be sufficiently similar, which is why the RBFE method is frequently employed to find the more potent molecules in a congeneric series during the lead optimization stage.^2,4^

The RBFE method has been historically hindered by a large computational cost and a series of practical challenges. These comprised, to name a few, system preparation, molecule superimposition (the part during which the alchemical path between two molecules is defined), whether the transformation can be validly computed, and performing molecular dynamics (MD) simulations with sufficient sampling. Over the past several years, significant improvements have been made in software,^5−9^ hardware costs,^10,11^ and sampling methodologies,^12,13^ increasing the feasibility of RBFE calculations. It is worth stating that there is considerable room for improvement involving the treatment of protonation states or isomerism, uncertainty quantification,^14^ as well as the generation and selection of the most promising ligands.

RBFE approaches are largely divided into two groups, free energy perturbation (FEP) and thermodynamic integration (TI). Whereas both calculate the energy differences along the alchemical path, the latter integrates over the derivative of the potential energy with respect to the control variable (λ). RBFE calculations are supported by the majority of MD engines, such as AMBER,^15^ CHARMM,^16^ and GROMACS^17^ to name a few. However, the full protocols require additional functionalities. The FEP protocol, among others, is implemented in packages including the proprietary FEP+ by Schrödinger,^4,5^ the open source package pmx^12,18^ based on GROMACS,^17^ and TIES,^7,19^ which also supports TI, and which is currently based on NAMD^20^ and OpenMM.^19^ Online services for input preparation include FEPrepare^21^ with support for NAMD with the OPLS-AA forcefield, CHARMM-GUI^22,23^ supporting NAMD, GENESIS,^24^ and the AMBER engine with GPU support for TI.^25^ It is worth pointing out that FEP and TI have been shown to produce equivalent results providing that a robust ensemble sampling is employed.^19^

In this paper we present a new release of the Python package TIES which is open source under the MIT license, along with a web portal ccs-ties.org interface to the software. We discuss the architecture of the software, provide some examples of its use, and conclude with a discussion of the planned developments.

## Theory

**Figure 1:**
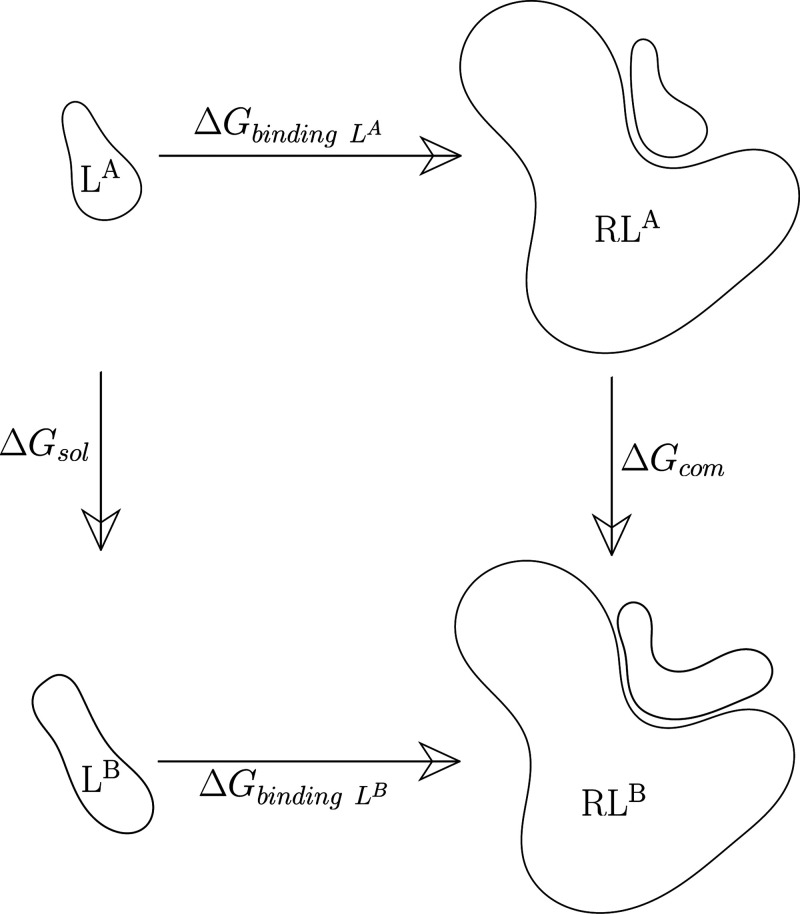
Thermodynamic cycle for the relative binding free energies (RBFEs) summarizing the relationship ΔΔ*G* = Δ*G*_*binding L*^*B*^_ – Δ*G*_*binding L*^*A*^_ = Δ*G*_*com*_ – Δ*G*_*sol*_. The horizontal paths for the calculation of the binding energy are slow due to the large difference between the end-states and the complex phase-space that separates them. The vertical path, referred to as the alchemical transformation, addresses this issue by mutating the ligand *L*^*A*^ into *L*^*B*^, largely reducing the size of the sampling space to the difference between the two ligands which, if sufficiently small, is computationally tractable.

Alchemical free energy methods invoke unphysical processes to estimate the FE changes between physical states (see Figure 1). The approach utilizes “alchemical” paths between thermodynamic end-states, providing a practical and efficient approach to predict the changes of thermodynamic properties. A series of alchemical intermediate states, represented by a coupling parameter (λ), are commonly introduced to connect the thermodynamic end-states. These λ states do not exist in real chemical space but can be validly modeled within computers. Since the FE is a function of state it does not matter which path is followed as long as the end-states are physical.

Here we focus on one application of the alchemical methods—the relative binding free energies—which calculates the difference in the binding free energy across two ligands in a protein receptor. Alchemical intermediate states are engineered to connect the physical states—ligand bound and unbound—with a protein target. Multiple intermediate λ states are needed to ensure neighboring states are sufficiently overlapped and hence provide a smooth connection between the two end physical states. This overlap is necessary in order to correctly estimate the energy differences (and their integral) along the states. Once the alchemical process has been fully performed, RBFEs can be estimated by multiple schemes^2^ such as TI,^26^ FEP,^27^ Bennet acceptance ratio (BAR),^28^ or multistate BAR (MBAR).^29^ The latter methods can be applied retrospectively to simulations that employed the TI scheme.

TI uses the derivative of the potential energy (*V*) with respect to the control variable λ. To calculate the difference in free energy *G*, integration over the control variable takes place, using an ensemble at each λ:
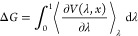
1where *x* is the coordinate and λ represents an ensemble of the converged states, with the RBFE value defined as
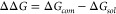
2

Scaling the Lennard-Jones potential to low values can create numerical instabilities when the distance to other atoms approaches zero. This is addressed by employing a soft-core potential which “limits” the values to computationally tractable ones. The slightly different variants of a soft-core potential employed in OpenMM and NAMD are described in detail in a previous publication.^19^

Two types of topologies are commonly used in RBFE calculations: single and dual topologies. In a single topology, a transformation is set up to involve minimal non-interacting dummy atoms, while some atoms may appear at both the initial and final states but with different atom types and properties. In contrast, no atoms are allowed to change types or parameters in a dual topology. Atoms appearing in only one state become dummy in another state. For simulations with any of the two topologies, those dummy atoms are partially interacting with their environment in the intermediate states, while the strengths of the interactions are scaled by the coupling parameter λ. For the single topology, those atoms mapped between two end-states are fused in the intermediate states; they present mixed properties partially from one end-state and partially from the other end-state. The use of dummy atoms in dual topology methods can be advantageous in avoiding the ring-breaking problems that can be encountered in single topology methods. This approach avoids the ring-breaking problems that are encountered in the single topology.^30,31^

The TIES protocol^32^ performs ensemble simulations at each λ-window. Ensemble averaging is performed to obtain the averages for the derivative of the potential energy with respect to λ, ∂V/∂λ. A stochastic integration method is then applied to get the FE changes of the process with λ changing from 0 to 1 or vice versa.^32^ Ensemble averaging provides us with the means to quantify uncertainty and hence to control errors. The convergence of TIES calculations can be evaluated by the differences introduced by adding another replica to the ensemble.^7^ There is a trade-off between the size of uncertainties and the associated computational cost. It should be noted that although TI is commonly applied in TIES, other FE approaches can be implemented as well, as we have done with FEP^19^ and MBAR^14,32,33^ on the condition that appropriate sampling is carried out.

## TIES 2.0 Software

TIES 2.0 is a Python package for the preparation of the input for the RBFE calculation and has been described in detail previously.^7^ The package, at its core, comprises four stages: 1) AM1-BCC charge assignment and GAFF parameterization of the ligand; 2) mapping of the ligand–ligand transformation using the dual-topology approach; 3) adjustment to the charges that ensures equal net charges in the alchemical regions; and finally 4) generation of the input files for the thermodynamic integration of MD simulations.

The AmberTools software^34^ is employed for the computation of the AM1-BCC charges and for GAFF (version 2) parameterization. The ligand-to-ligand transformation mapping uses an in-house-developed tailored superimposition algorithm in which the ligands are jointly traversed to generate the maximum common substructure (MCS).^7^ The charges between the matching atoms in the common area of the two ligands are averaged, and any introduced imbalance is equally distributed across the alchemical region.

The user can interact with TIES 2.0 either via the application programming interface (API) or via a command line interface. The latter is added for convenience and is itself implemented with the API.

TIES 2.0 has a modular design, and the quality and status of the implementation are continually verified using a combination of unit tests and continuous integration. The software, along with the TIES protocol, was initially validated in the calculation of 55 alchemical transformations, with comparisons made against the previous implementation,^7^ and has since been used extensively in other projects.^7,19,32,35^

TIES 2.0 follows the *principle of least astonishment* rule of thumb, which states that the behavior of the software should strive not to astonish its users. In this case, a set of validated defaults, as described in the TIES protocol, are assumed.^7,32^ These include, for example, the cutoff value of 0.1e to decide whether two superimposed atoms of the same type should be assigned to a joint area of the alchemical transformation, or disallowing the molecule to be divided by an alchemical area. These defaults are controlled by a single configurable module, *Config*, which can then be passed to other TIES modules. This central configuration streamlines the creation of new workflows, making it suitable for application on a congeneric series.

The basic building block behind any alchemical transformation is the definition of the transformation between two ligands. Defining this transformation takes place in the *Pair* module, which requires at least two ligands and their net charge as input. The transformation can be prepared with a few lines of Python (lines 1–5, Figure S1). This example can be easily extended to execute the MD simulations with the TIES_MD package, with three more lines needed to complete the TIES RBFE protocol (lines 6–8, Figure S1).

To extend Figure S1, run the MD simulation, and perform the analysis, the four lines of code outlined in Figure S2 must be added. The example for running MD simulations given in Figure S2 would work on a GPU workstation or while using high-performance computing (HPC) interactively. For large job sizes neither local workstations nor interactive use is appropriate. An example of how to use TIES on supercomputers will be presented in a later section.

**Figure 2:**
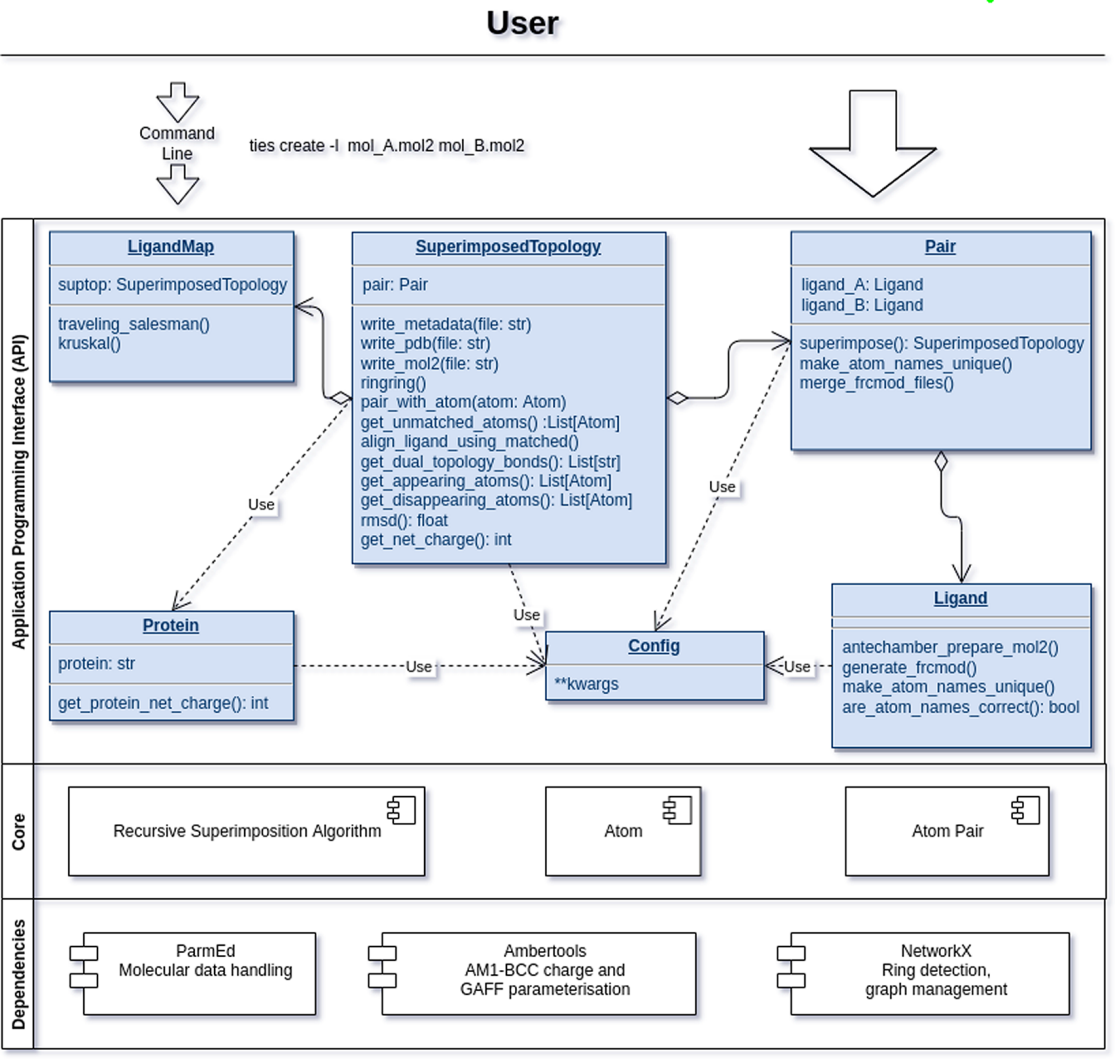
Architecture of the TIES 2.0 software. An alchemical transformation is represented by the *SuperimposedTopology* class which outputs the alchemical path for any two ligands. The *Config* module centrally controls the configuration of other modules and by default employs the established TIES workflow.

The full set of provided modules is presented in Figure 2. At the top we highlight how the user can access TIES via the command line or the API directly. The top container (API) presents the modules exposed to the user via the Python API. The *Pair* class facilitates the preparation of a transformation, which in turn is represented by the class *SuperimposedTopology*. The middle container, Core, contains the basic helper classes along with the recursive superimposition algorithm, which finds the MCS in the alchemical transformation.

The superimposition algorithm invokes a recursive joint traversal across two given ligands to find the largest overlap. Whereas a full search for the MCS is possible, it is very rarely necessary for computing the optimal MCS. Thus, the algorithm uses a heuristic which reduces the number of traversals to √*n*, where *n* is the average number of atoms in the smaller ligand. The selected pairs include heavy atoms that are of a rare type, common to both ligands, and preferably reside outside of any aromatic rings.

The complete API documentation is made available on the TIES web portal (ccs-ties.org).

## WebTIES Portal

WebTIES is a web portal that facilitates the use of the TIES 2.0 software, and therefore further simplifies the preparation of files for RBFE calculations. The server side carries out charge assignment, parameterization, and transformation preparation, as well as providing limited storage for each user. By deploying web access to the TIES protocol we further reduce the barriers to the design of RBFE studies.

We enable the user to access the TIES package functionality and protocol without the need of installing any software. In the first version of the web portal the well-established TIES protocol is used.^32^ Two file formats are supported: PDB and MOL2. If the MOL2 format is used and the ligands already contain charges, these will be used instead of computing the AM1-BCC charges. In the current implementation of the service, the ligands are parameterized with the General Amber Force Field (GAFF). The protein can be also added to the system. Users can manually select any two ligands and generate a hybrid transformation. The transformations are then queued for processing and the generated files made available for download.

The files generated on the portal can be used directly as input for the TIES_MD package which can either utilize OpenMM or NAMD (2.14, 3+) as the molecular dynamics engine (see Simulation Engines). The log and the history of the operations is kept for the user to investigate. This workflow is shown in Figure S3, which presents the use of the website. Finally, the two complementing open source packages TIES_MD and ties_ana allow one to execute the RBFE calculations and perform analysis on output data, therefore completing the TIES protocol.

**Figure 3:**
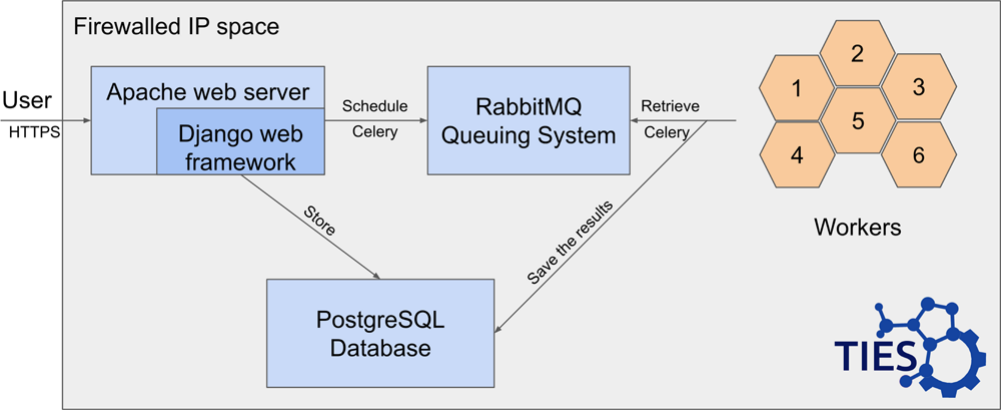
Server side of the WebTIES architecture. The queuing system (RabbitMQ) together with the attached workers is used to manage the load of user-submitted jobs. The *celery* package is utilized for the communication with the queuing system over the advanced message queuing protocol (AMQP).

The WebTIES software on the server side ensures that any scheduled work will not overstretch the resources dedicated to the service. This is done by employing a queuing system for processing, such as charge assignment with the AM1-BCC scheme or generation of the mapping between two ligands. A list of workers monitor the queue for any work and, after processing, submit the results back to the database. The arrangement is presented in Figure 3. As the web server, the Django framework was used along with a PostgreSQL database. The communication with the database is carried out using an object relational mapping (ORM). A request to generate an alchemical transformation is added to the queuing system via the *celery* package which handles the Advanced Message Queuing Protocol (AMQP). RabbitMQ^36^ was used as the queuing system (AMQP implementation). On a separate set of nodes, workers wait for any jobs from the queuing system similarly utilizing the *celery* package.^37^ Once a job is processed, it is then updated in the database by the worker, which in turn triggers a notification for the user of the completed job.

The communication between the Internet browser and the WebTIES server is handled using the representational state transfer (REST) protocol. The approach further decouples the client from the server, permitting WebTIES to provide external services. In a prospective example, a user designs a congeneric series on the WebTIES portal, followed by a employing a thin Python client on a supercomputer to download and execute the jobs in an automated manner. This approach is automated while being entirely agnostic to the HPC hardware.

The RabbitMQ queuing system is inherently scalable, enabling an easy addition of workers. Whereas these workers are currently hidden behind a firewall, it is possible to similarly attach them in the future to the secure REST interface, thereby allowing for a more versatile computing paradigm.

## Simulation Engines

TIES 2.0 and TIES_MD are constructed to be agnostic to which molecular dynamics engine is used to perform the simulation. Currently we offer support for OpenMM and NAMD. Owing to differences between OpenMM and NAMD and the different ethos of each code, our support for these engines also differs in implementation.

OpenMM is constructed as a library with a full Python API. As such TIES_MD is tightly integrated with OpenMM and can perform all setup and simulation steps without external calls to the command line. NAMD does not provide such an API, and thus TIES_MD will write intermediate NAMD configuration scripts to disk, which can then be run via NAMD on the command line or via a batch scheduler on a high-performance computer. Example HPC submission scripts are also written by TIES_MD to aid the user in running their simulations.

Minor differences in the NAMD and OpenMM alchemical protocols exist in TIES_MD. These differences are caused by the lack of perfect feature parity between the two codes. Our validation of TIES_MD has shown that these differences do not manifest significantly in the result and that the aleatoric error stemming from the inherently chaotic nature of MD trajectories is the dominating source of uncertainty in these RBFE calculations.^19^

Both OpenMM and NAMD (2.14, 3+) engines offer support for CPU and GPU systems. The new versions of NAMD (3+) provide performance competitive with OpenMM on modern GPU hardware. Typically each RBFE simulation uses 13 alchemical windows and 5 replicas in each window.^32^ This results in 65 simulations which can be easily parallelized over 65 GPUs, with each GPU running an independent simulation. Using 65 NVIDIA V100 GPU, TIES_MD can calculate one RBFE for a system with 35 000 atoms in approximately 75 min using either OpenMM 7.7.0 or NAMD 3. This is around 5 times faster than a CPU-based calculation using 6240 cores.

## High-Performance Computing Example Application

In this section we give a brief overview of a TIES application in real-world scientific research. Molecule fluorination is employed extensively for improving the desired physicochemical properties.^38,39^ We run a fluorine scanning calculation which iterates over all hydrogen atoms bonded to a carbon in a molecule and substitutes them one at a time for fluorine.^40^ The inputs for these simulations can be set up with TIES 2.0 as in Figure S1. TIES_MD can then run the calculation as shown in Figure S4.

This will produce a submission script for each fluorinated analogue which can be submitted to the supercomputer’s scheduler. The submission script will automatically iterate over all the different MD simulations and assign one GPU per simulation. We provide one full submission script generated by the above code in Figure S6. For one molecule with 24 fluorinations this results in 18 720 separate MD simulations.

For demonstration purposes we have applied this method to a factor Xa inhibitor (Figure S5) known to be susceptible to a fluorination that beneficially improves the binding affinity. Figure S7 shows the 2D structure of the input drug and best fluorinated analogue found experimentally in previous work.^41^

When we apply TIES to this fluorine scanning problem the results are a ΔΔ*G* and associated standard error of the mean for each fluorination. The full results for the calculated ΔΔ*G*’s can be found in Table S1. The 3D structure of the factor Xa inhibitor, colored by the ΔΔ*G* of the tested fluorinations, is visualized in Figure S8. The highlighted hydrogen H18 is identified as the best position for fluorination in agreement with the previous computational work.^40^ The experimental work also pointed to this fluorination site, which improved the inhibition potency 60-fold.^42^

## Conclusions

Relative binding free energies have emerged as one of the leading computational approaches in obtaining accurate binding free energies.^43^ However, the inherent complexity in the process translates to a steep learning curve that can incur significant costs in time and effort.

Our software described in this paper addresses and ameliorates this issue, through our release of the open source package TIES 2.0 as well as a web portal called WebTIES.

The TIES 2.0 software is built upon the well-established TIES protocol. Together with the TIES_MD package, and with its Python API, TIES 2.0 allows for rapid development and easier adaptation of RBFE calculations. The TIES 2.0 software can be combined with the available package TIES_MD for running MD simulations. It supports OpenMM and NAMD (2.14, 3+) molecular dynamics engines for the computationally demanding component of the alchemical relative FE calculations.

WebTIES facilitates the preparation of dual-topology alchemical transformations and can be used in place of TIES 2.0. We described the architecture of the web portal and demonstrated the attainment of two important goals. First, scalability is achieved via a queuing system for processing, which enables the automated addition and removal of workers. The second is the decoupling achieved by employing the representational state transfer (REST) for the communication in WebTIES and a client. Whereas in this iteration the client means an Internet browser, the separation will allow a user to login to WebTIES from a HPC cluster and directly execute the RBFE calculations.

With the scalable infrastructure and decoupled interface, we are en route to performing remote computation. The ultimate aim is the execution of remote computational jobs in which alchemical transformation calculations are spawned directly from the web portal. Programming libraries that understand the high-performance cluster-specific software already exist.^44^ Furthermore, limited implementations, in which the computationally heavy calculations are offloaded to a cluster, have been implemented before (HemeLB-HOFF^45^). Therefore, in this release we have laid the basis for a fully automated “one-stop-shop” with the intention of enabling the user to obtain high-quality binding energies with minimal effort or technical skill.

By providing a full and validated pipeline for biomedical research, we further enable users to focus on their scientific interests, rather than the many technical issues which can beset them. Further automation of the process will increase the potential user base, an important factor in the ever more expensive search for novel medicines. As we witness further improvements in the workflows,^46,47^ automated and systematic forcefield development,^48^ computational costs,^11^ targets and their variations,^49,50^ together with accessibility and automation, we expect to encounter more real-life applications and successes.

## Data Availability

TIES 2.0 is available at https://github.com/UCL-CCS/TIES. TIES_MD is available at https://ucl-ccs.github.io/TIES_MD. WebTIES can be accessed at https://ccs-ties.org/.

## Supporting Information Available

The visualized molecule used in the fluorination study case, with labeled hydrogens; tabulated results of fluorination; and an example of a submission script for the Summit supercomputer (PDF)
