# Supplementary material for: TIES 2.0: A Dual-Topology Open Source Relative Binding Free Energy Builder with Web Portal
Source: J Chem Inf Model. 2023 Jan 31;63(3):718–24. doi: 10.1021/acs.jcim.2c01596 (PMC9930115; doi:10.1021/acs.jcim.2c01596)
Supplement: Supplementary file 1 — ci2c01596_si_001.pdf [file ci2c01596_si_001.pdf]

Supporting Information: TIES 2.0: a Dual-Topology Open Source Relative Binding Free Energy Builder with Web Portal

Mateusz K. Bieniek,[†,‡] Alexander D. Wade,[†] Agastya P. Bhati,[†] Shunzhou Wan,[†] and Peter V. Coveney[*,†,¶,§]

†*Centre for Computational Science, Department of Chemistry, University College WC1H 0AJ, United Kingdom*

‡*School of Natural and Environmental Sciences, Newcastle University, Newcastle upon Tyne NE1 7RU, United Kingdom*

¶*Advanced Research Computing Centre, University College London, London WC1H 0AJ, United Kingdom*

§*Institute for Informatics, Faculty of Science, University of Amsterdam, 1098XH Amsterdam, The Netherlands*

E-mail: p.v.coveney@ucl.ac.uk

Here we present the supplementary information for the paper TIES 2.0: a Dual-Topology Open Source Relative Binding Free Energy Builder with Web Portal. Contained in this SI is a full presentation of the binding free energy results from a fluorine scanning calculation performed with TIES 2.0. These binding free energy results are presented in Table S1 and Figure S5. Also in this SI we show in Figure S6 one full submission script generated by TIES 2.0 which was used to calculate these fluorine scanning results on Summit, a GPU-accelerated supercomputer at the Oak Ridge National Lab.

S1 TIES 2.0 Software

```
1.from ties import Pair, Config, Protein, MD

# simulation settings
2.config = Config(workdir="ties2", md_engine="openmm", protein="protein.
                                                    pdb")

# load two ligands and create a hybrid
3.pair = Pair("lig1.mol2", "lig2.mol2", config)
4.pair.make_atom_names_unique()
5.hybrid = pair.superimpose()

# set up ligand simulation
6.hybrid.prepare_inputs()

# add protein and set up complex simulation
7.protein = Protein(config=config)
8.hybrid.prepare_inputs(protein=protein)
```

Figure S1: TIES 2.0 python code used to generate the superimposition and input files for RBFE calculations.

```
# run ligand and complex simulations
1.for leg in ["lig", "com"]:
2.    simulation = MD(f"ties2/ties-lig1-lig2/{leg}")
3.    simulation.run()

# run the analysis
4.simulation.analysis(legs=["lig", "com"])
```

Figure S2: TIES_MD is used to generate and perform the molecular dynamics simulations and free energy analysis.

S2 WebTIES Portal

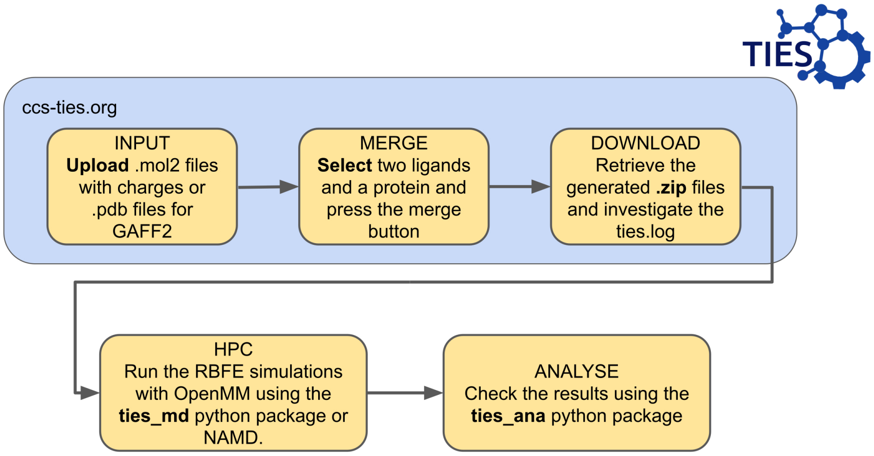

Figure S3: The workflow implemented in the WebTIES portal (blue container) and its place in the relative binding free energy preparations. The generated files can be used as the input for the ties_md package to employ OpenMM or NAMD as the molecular dynamics engines.

S3 High Performance Computing Example Application

```
# loop over the names of fluorinated molecules created with TIES 2.0
for molecule in fluorinated_molecules:
    for leg in ["com", "lig"]:
        simulation = MD(f"{molecule}/ties-ligA-{molecule}/{leg}")
        # change the header of generated submission scripts
        simulation.options.sub_header = "#BSUB -W 120 \n#BSUB -nnodes 13
        module load cuda """
        # supercomputer specific element of the run line
        simulation.options.pre_run_line = "jsrun -n 1"
        # configure the ties_md run line
        simulation.options.run_line = "ties_md --rep_id=$i
        --config_file=$ties_dir/TIES.cfg
        --windows_mask=$lambda, $(expr  $lambda + 1) "
        # setup output directories and submission scripts
        simulation.setup()
```

Figure S4: Setting up simulations of fluorinated analogues for an arbitrary supercomputer. Here we target Summit at Oak Ridge National Laboratory.

Table S1: TIES results from fluorine scanning ($\Delta\Delta G$ and associate standard error of the mean).

ligand	ddG [kcal/mol]	SEM	EXP [kcal/mol]
l_H18	-2.76	0.03	-2.37
l_H15	-1.04	1.12	
l_H9	-1.03	0.05	
l_H6	-0.9	0.03	
l_H29	-0.88	0.06	
l_H7	-0.83	0.05	
l_H19	-0.47	0.04	
l_H26	-0.36	0.04	
l_H21	-0.17	0.03	
l_H23	-0.1	0.06	
l_H12	-0.07	0.03	
l_H24	-0.05	0.05	
l_H13	-0.03	0.03	
l_H3	-0.03	0.02	
l_H25	0.11	0.04	
l_H22	0.17	0.04	
l_H8	0.64	0.04	
l_H4	0.65	0.09	
l_H28	0.92	0.04	
l_H20	1.52	0.11	
l_H5	1.81	0.12	
l_H16	2.38	0.06	
l_H14	3.86	0.83	

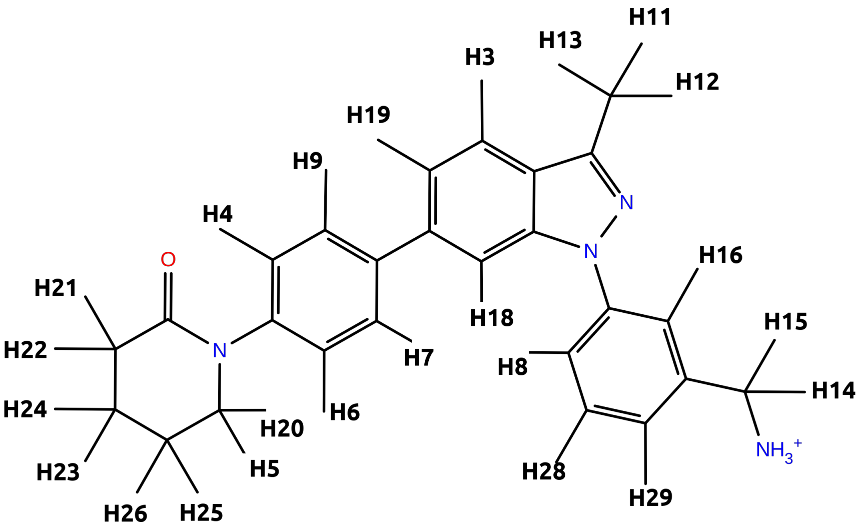

Figure S5: The molecule on which the fluorination scanning was performed as described in the main paper. All of the hydrogens are labelled.

```
#!/bin/bash
#BSUB -P projectID
#BSUB -W 120
#BSUB -nnodes 13
#BSUB -alloc_flags "gpudefault smt1"

module load cuda

export ties_dir="/absolute_path/ties-ligA-l_H18/com"
cd $ties_dir

for lambda in {0..12}; do
    for i in {0..5}; do
        jsrun --smpiargs="off" -n 1 -a 1 -c 1 -g 1 -b packed:1
        ties_md --windows_mask=$lambda,$(expr $lambda + 1) \
            --rep_id=$i > $ties_dir/$lambda$i.out&
    done
done

wait
```

Figure S6: Example of a submission script for the Summit supercomputer using the OpenMM molecular dynamics engine.

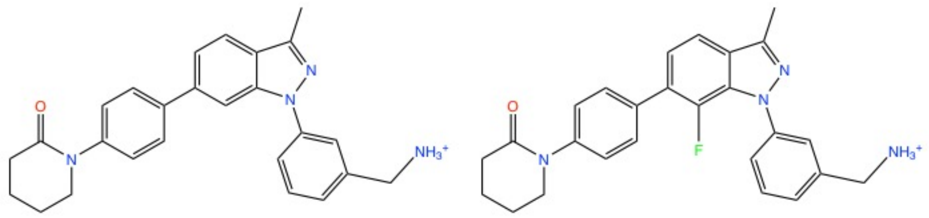

1

2

Factor Xa K_i = 6850 nM

Factor Xa K_i = 124 nM

Figure S7: Panel 1 shows a factor Xa inhibitor and its inhibition coefficient. Panel 2 shows a fluorinated analogue of the inhibitor in panel 1 with a 55 fold increase in the inhibition coefficient.[1]

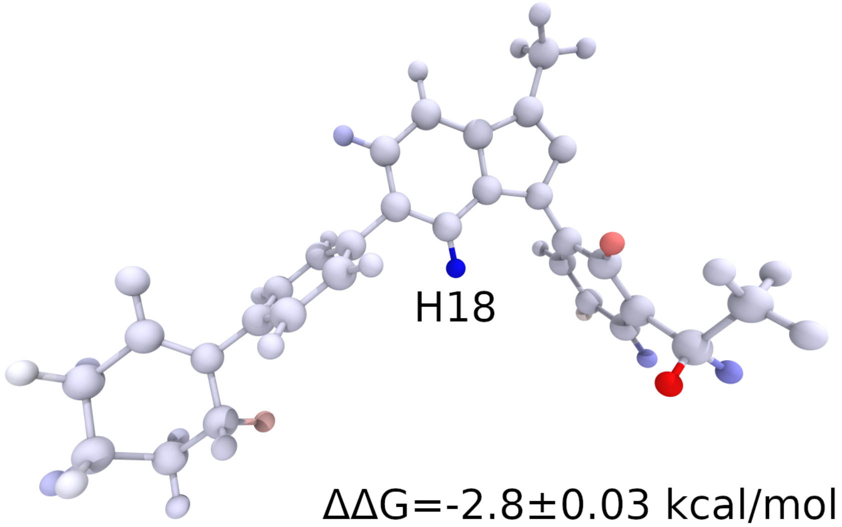

Figure S8: The results of the simulations set up in Figure S4. Atoms are coloured by the $\Delta\Delta G$ value calculated for substituting that atom with a fluorine atom (only hydrogen bound to carbon is substituted). Blue denotes a more negative $\Delta\Delta G$ and red a more positive value. H18 is the most negative (-2.8 kcal/mol) in agreement with the experimental findings.[1]

References

(1) Lee, Y.-K.; Parks, D. J.; Lu, T.; Thieu, T. V.; Markotan, T.; Pan, W.; McComsey, D. F.; Milkiewicz, K. L.; Crysler, C. S.; Ninan, N.; Abad, M. C.; Giardino, E. C.; Maryanoff, B. E.; Damiano, B. P.; Player, M. R. 7-Fluoroindazoles as Potent and Selective Inhibitors of Factor Xa. *Journal of Medicinal Chemistry* **2008**, *51*, 282–297, PMID: 18159923.
